# Musk Tongxin Dripping Pills for treating Ticagrelor in Patients After Percutaneous Coronary Intervention: Echocardiography Combined with Untargeted Metabolomics

**DOI:** 10.3389/fphar.2021.731734

**Published:** 2021-10-27

**Authors:** Lyu Nan, Lai Peng, Zhao Jinxia, Guo Mengzhe, Liang Jun, Wang Haibo, Geng Houfa

**Affiliations:** ^1^ Clinical College, Xuzhou Medical University, Xuzhou Central Hospital, Xuzhou, China; ^2^ Jiangsu Key Laboratory of New Drug Research and Clinical Pharmacy, Xuzhou Medical University, Xuzhou, China

**Keywords:** Musk Tongxin Dripping Pill, ticagrelor, echocardiography, untargeted metabolomics, LC-MS

## Abstract

**Objectives:** As current clinical practice guidelines, ticagrelor is the suggested therapeutic scheme to prevent adverse cardiovascular events in acute myocardial infarction (AMI) patients undergoing percutaneous coronary intervention (PCI) treatment. However, this therapeutic strategy still fails, and around 30% patients display inadequate antiplatelet responses. Musk Tongxin Dripping Pill (MTDP) in Chinese hospital was usually considered as the combination with ticagrelor to improve the treatment effect. Unfortunately, the mechanism has not been elucidated.

**Methods:** The untargeted metabolomic method was introduced based on liquid chromatography–high-resolution mass spectrometry (HPLC-HRMS) coupled with STI for the research of the drug combination mechanism between ticagrelor and MTDP. 28 patients with a confirmed diagnosis of AMI were selectively collected, who were then divided into two different dosage regimen groups, and the serum samples were collected for the untargeted metabolomics assay. Then the differential metabolites were associated with blood biochemical indicators.

**Results:** The GLS values in both groups increased after treatment and those in the ticagrelor and MTDP combination group after treatment were higher than those in the ticagrelor group (*p* < 0.05), suggesting that the combination medication has better therapeutic effect on patients with myocardial infarction. From metabolomics analysis, the species of metabolites changed in two groups before and after treatment. Moreover, 93 differential metabolites changed in the drug combination group compared with the ticagrelor group after treatment (*p* < 0.05), which mainly related to changes in fatty acid metabolism pathways. Then the differential metabolites were found to be related with blood biochemical indicators, such as lipid, high-density lipoprotein (HDL), and low-density lipoprotein (LDL).

**Conclusion:** This work will provide a possible mechanism of the drug combination interaction between ticagrelor and MTDP from two angles of echocardiography and metabonomics. Several potential metabolic pathways were also found to have a relationship with MTDP, which will provide a new perspective in clinical medication.

## Introduction

Acute myocardial infarction (AMI) is myocardial necrosis caused by acute and persistent ischemia and hypoxia of the coronary artery (Liam et al., 2021). As the primary cardiovascular disease, AMI seriously affects health and life safety of human beings, which causes over 30% mortality of coronary heart disease patients (Blais et al., 2020; Thomas et al., 2020). Percutaneous coronary intervention (PCI) is the guideline-recommended treatment for patients undergoing ST-segment elevation AMI (Matthews and Frishman, 2017). As the development of this technique, the PCI is now considered safe with the decrease of periprocedural complications, including the rates of associated stent thrombosis, Q-wave myocardial infarction, stroke, and death (Stephen et al., 2014). However, frequent periprocedural myonecrosis can still be found through highly sensitive cardiac troponin assay (Reed et al., 2017). Although these periprocedural complications were often asymptomatic, they can delay hospital discharge and have been associated with an increased risk of future major cardiac adverse events, containing death (Warren-Gash et al., 2009). Therefore, the patients also need antiplatelet therapy after surgery, which related to the prognosis of the patients.

At present, clopidogrel and ticagrelor are the two most commonly used antiplatelet agents in clinic (Johanne et al., 2020; Georg et al., 2021). Dishearteningly, despite the greater preponderance of ticagrelor, this treatment is still not ideal, with around 30% of patients showing an inadequate antiplatelet response (Lee et al., 2020). Therefore, in modern clinical practice of China, several drugs were considered as the combination with ticagrelor for the therapy. In particular, traditional Chinese medicine (TCM) usually used to be a significant adjuvant therapy jointly with ticagrelor. Musk Tongxin Dropping Pill (MTDP) is one of the most commonly used compound Chinese patent medicines in combination with ticagrelor (Liu et al., 2021; Zhao et al., 2021). The ingredients of MTDP mainly contain *Salvia miltiorrhiza* Bunge (Lamiaceae; *Salviae miltiorrhizae radix et rhizoma*), Artificial *moschus*, *Panax ginseng* C.A.Mey (Araliaceae; *Panax quinquefolius Linnaeus* var. *ginseng*), *Venenum bufonis*, artificial *Bovis calculus*, and *Borneolum* (Dipterocarpaceae; *Dryobalanops aromatica* Gaertn.f.), which have the curative effect in the elimination of blood stasis, the promotion of blood circulation, and the dredging of collaterals. Additionally, MTDP has also been reported to increase coronary blood flow, and improve myocardial hypoxia and ischemia tolerance, which can help to reduce the scope of myocardial injury. So far, the combination of ticagrelor and MTDP was extensively used in the department of cardiology, Xuzhou central hospital, which achieved good therapeutic effects. Some other literatures also reported the positive clinical effects of this combination therapy in improving efficacy and reducing adverse reactions. However, there are few studies on the pharmacodynamic mechanism and drug–drug interaction mechanism. One of the main reasons is the lack of appropriate research methods. Therefore, new strategy must be progressed to study the synergistic effect between ticagrelor and MTDP.

Metabolomics is an emerging method, which is considered to be a comprehensive and synchronous analytical method for the identification and quantification of small-molecule metabolites, and their amount changes under different circumstances (Chen et al., 2010; Basak et al., 2015a). In this way, the characteristics of biological systems can be objectively measured and evaluated, and can be used as indicators to be effective in the reciprocity between biological systems and intervention. Therefore, it will provide possibilities for the integral analysis of drug combination to explore the pharmacodynamic mechanisms. In addition, the un-targeted metabolomic strategy provides fast and high-flux assay of abundant possible metabolites in a given sample without the knowledge of metabolites in advance, which is appropriate for the study of drug combination mechanisms (Hua et al., 2011; Duranton et al., 2014). For un-targeted metabonomic applications, a variety of detecting platforms can be used, such as high-performance liquid chromatography–mass spectrometry (HPLC-MS), gas chromatography–mass spectrometry (GC-MS), and nuclear magnetic resonance (NMR). Among all these platforms, mass spectrometry has high sensitivity, high accuracy, high flux, minimal sample preprocessing requirements, and high data quantitation and reproducibility. In particular, the high-resolution mass spectrometry (HRMS) techniques provide accurate molecular weights, which can be used for the investigation of molecular formulas and structures (Yang et al., 2014; Ju et al., 2015). In addition, an association of separation strategy, containing SPE, GC, or HPLC, can reduce the complexity of the mass spectrometer, helping the suggestion of additional information on the metabolites’ chemical properties.

Accurately assessing the cardiac function changes can help to evaluate the therapeutic effect of patients, as well as investigating the mechanism of drug combination. The speckle-tracking imaging (STI) technique, based on echocardiography, can delineate the region of interest (ROI) on the two-dimensional image of the cardiac wall. It will track the movement of the echo spots frame by frame automatically as the cardiac cycle in this ROI, which can calculate the strain of myocardial tissue by measuring the displacement and evaluate the systolic and diastolic functions of the myocardium. The strain of myocardial tissue, representing the various stages of myocardial deformation, contains longitudinal deformation, radial deformation, circumferential deformation, and rotational deformation. In particular, longitudinal deformation myocardium accounts for 70% of the total myocardium, which is more susceptible to ischemic damage when coronary artery stenosis is experienced. Therefore, longitudinal deformation would reflect the myocardial systolic function more sensitively.

Herein, the mechanism of ticagrelor and MTDP combination was studied by un-targeted metabonomics based on HPLC-HRMS combined with STI. Serum analysis was performed before and after treatment in two groups, including the ticagrelor group and the ticagrelor and MTDP combination group. The STI technique was used to evaluate the myocardial function. The metabolite content changes were accurately detected to elucidate the combination mechanism between ticagrelor and MTDP. This study will make a basis for the clinical drug combination between TCM and western medicines.

## Materials and Methods

### Pharmaceutical Composition Analysis

The composition of MTDP has been analyzed by using liquid chromatography–high-resolution mass spectrometry. First, the paraffin shell of the pill was stripped. Then the drug components were extracted by two solvents, methanol and dichloromethane. Next, the extractive was dried at low temperature and redissolved by methanol. Then the solution was analyzed by liquid chromatography–high-resolution mass spectrometry.

*LC-MS conditions*: LC separation was carried out by Waters e2695 Ultra-HPLC (Waters, United States) with a Fortis type of C18 column (4.6 × 150 mm, 3.5 μm). The temperature of the column was setup at 40°C. Solvent A of the mobile phase was water/acetic acid (99.9/0.1, *v/v*), and solvent B was methanol. The gradient was 0 min, 5% (B) to 20 min, and 95% (B). The flow rate was 0.3 ml min^−1^, and the injection volume was 5 μl. Positive and negative modes of mass spectrometric assay were performed on Thermo Velos mass spectrometer (Thermo, CA, United States). A full scan mode was used for detection.

### Study Subjects

Patients with cancer, bleeding, gastrointestinal hemorrhage, severe heart exhaustion, significantly abnormal clinical test indicators, and high sensitivity to ticagrelor or MTDP were excluded. 28 patients with a confirmed AMI were enrolled, ranging in age from 45 to 80 years. The clinical indicators of patients included the elevation of the ST segment and serum troponin levels. The patients with atrial fibrillation after the PCI were also excluded.

All subjects were informed with the benefits and risks of participating in the study by experienced physicians and voluntarily submitted in-person signed informed consents prior to participating in the clinical trial. The design and implementation of the study protocol were approved and supervised by the Ethics Review Committee of Xuzhou Central Hospital in accordance with the Declaration of Helsinki.

### Study Design

28 patients were randomly divided into two groups: 1) ticagrelor group (*n* = 14), and 2) ticagrelor and MTDP combination group (*n* = 14). All of them were given the PCI as the initial treatment. The dose of ticagrelor was 90 mg bid and that of MTDP was 70 mg tid. Aspirin and atorvastatin were used as basic treatment, and the dosages were 100 mg qd and 40 mg qd, respectively. Serum samples were collected according to the group before and 30 days after medication. All serum samples were frozen at -80°C.

### STI Detection

In this work, global longitudinal strain (GLS) was used to evaluate the drug therapeutic effect after 30-day treatment. The ultrasonograph was Philips EPIQ 7c (Philips, Germany) coupled with S5-1 ultrasonic probe. Three views were detected, including the apical four-chamber view, apical three-chamber view, and apical two-chamber view in four cardiac cycles. A boundary between inner and outer membranes of the left ventricle was drawn by the system and divided into 17 segments. GLS values were obtained by longitudinal deformation curves corresponding to these 17 segments, which is negative at myocardium contraction and positive at myocardium diastole.

### Sample Detection

*Sample pretreatment*: The serum samples were prepared before being detected by high-performance liquid chromatography–high resolution mass spectrometry (HPLC-Thermo HFX MS). In this preparation, 1,200 µl of precooled methanol and acetonitrile (50/50, *v/v*) was added into 200 µl of the serum sample for the albumen precipitation. Then the mixture was centrifuged after standing at −20 C for 10 min, and the centrifugal condition was 12,000 g for 10 min. Finally, volatile drying was performed to the supernate by rotary evaporation. Then dry powder was redissolved by 50 µl of acetonitrile.

*LC-MS conditions*: LC separation was carried out on Waters e2695 Ultra-HPLC (Waters, the United States) with a Fortis type of C18 column (2.1 × 100 mm, 1.7 μm). The temperature of column was setup at 40 C. Solvent A of the mobile phase was water/acetic acid (99.9/0.1, *v/v*) and solvent B was methanol. The gradient was 0 min, 95% (A); 1 min, 75% (A); 3 min, 55% (A); 6 min, 5% (A); 12 min, 5% (A). The post time was 2 min. The flow rate was 0.3 ml min^−1^, and the injection volume was 5 μl. Positive and negative modes of mass spectrometric assay were performed on Thermo HFX mass spectrometer (Thermo, CA, United States). A full scan mode were used for detection. Solutions were indoctrinated into the ESI source at 0.3 ml min^−1^ with the following parameters: 4000 V of capillary, 12 L min^−1^ of drying gas, and 350°C of drying gas temperature. Nitrogen was used as atomizing and drying gas. In order to obtain the highest detection sensitivity, all MS conditions were optimized.

Quality control (QC) samples were made by mixing all patients’ serum. The QC samples were detected for 6 times to check system stability prior to sample analysis, and every 10 samples were injected during sample analysis.

### Safety

Adverse events were identified through relevant physical examinations and health-related questions asked throughout the study. Physical examination and clinical laboratory examination (hematology, blood biochemistry, urinalysis, and blood aggregation tests) are performed on the day before experimental drug therapy and within 48 h of the last dose.

### Data and Statistical Analysis

Thermo Data Analysis software was used for the deconvolution of the LC-MS spectrum, including baseline correction, processing noise, and peak alignment. Then the compound detection (CD) was used for a matrix formed by retention time, m/z, and strength was then normalized by compound detection (CD). Then the matrix was analyzed by partial least squares discriminant analysis (PLS-DA), principal component analysis (PCA), and orthogonal PLS-DA (OPLS-DA) to obtain the differential metabolites among each group. SPSS16.0 software was used to further compare the differential metabolites data by one-way ANOVA, and an independent sample *t* test was conducted (*p* < 0.05). In addition, mass spectrometry data for different metabolites were traced to metabolite pathways through the Genomes (KEGG) and Kyoto Encyclopedia of Genes.

## Results

**FIGURE 1:**
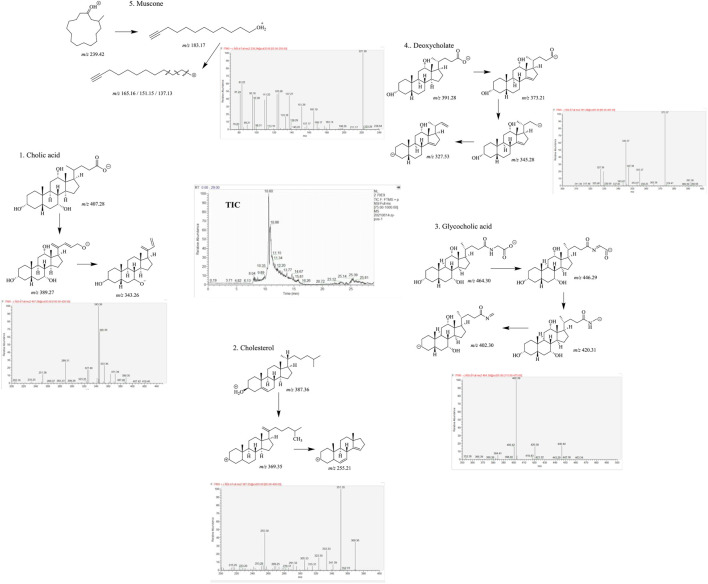
Five main components of MTDP by liquid chromatography–high-resolution mass spectrometry analysis.

From the high-resolution mass spectrometry analysis, five main compounds have been identified according to tandem mass spectrum, including cholic acid (parent ion *m/z* 407.28, sub-ion *m/z* 389.27, 343.26), cholesterol (parent ion *m/z* 387.36, sub-ion *m/z* 369.35, 255.21), glycocholic acid (parent ion *m/z* 464.30, sub-ion *m/z* 446.29, 420.31, 402.30), deoxycholate (parent ion *m/z* 391.28, sub-ion *m/z* 373.21, 345.28, 327.53), and muscone (parent *ion* m/z 239.42, sub-ion *m/z* 183.17, 165.16, 151.15, 137.13). We considered that these five compounds can act as the main pharmacodynamics components (as shown in Figure 1).

**FIGURE 2:**
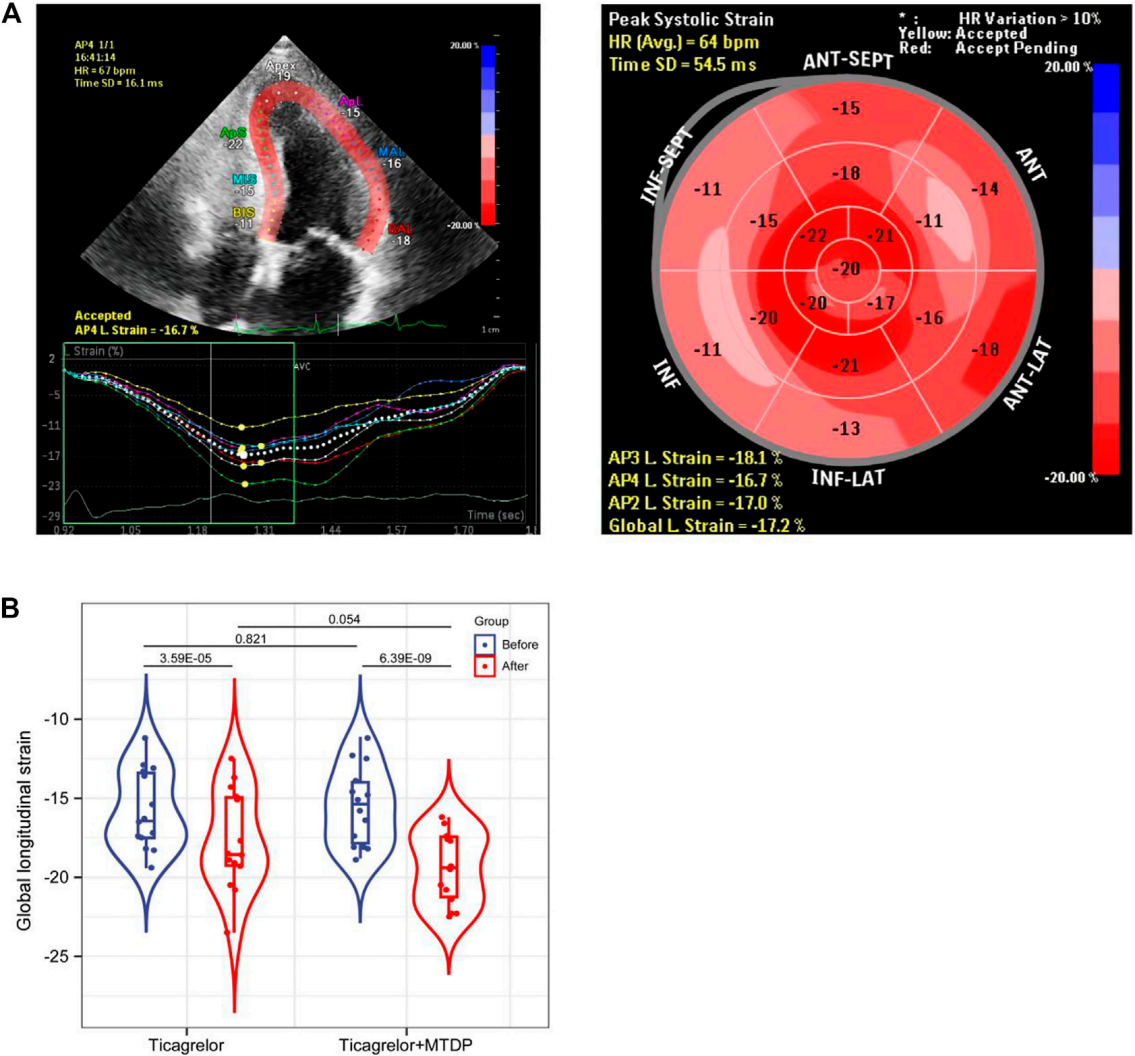
Comparison of GLS values between the ticagrelor group and the MTDP ticagrelor combination group by STI based on echocardiography.

28 patients were collected and divided into two groups: 1. ticagrelor group and 2. MTDP and ticagrelor combination group. The basic information of these patients can be found in Table 1. From the results, gender, age, and smoking status have no statistical differences between two groups. All these patients were treated with the same basic medication after the PCI. Moreover, the blood biochemical indicators of cardiac function, including CK-MB and LDH, have no statistical differences between two groups. After the drug treatment, the GLS values of patients were evaluated by STI. As shown in Figure 2, the GLS value in both groups increased after treatment, indicating that the medication can improve the prognosis of patients. In addition, the GLS values in the drug combination group after treatment were higher than those in the ticagrelor group, suggesting that the combination medication has better therapeutic effect on patients with myocardial infarction. The metabolomic results will elaborate the specific mechanism of the drug combination improving the therapeutic effect.

**TABLE 1:** Basic patient enrollment information.

Group	Drug combination group	Ticagrelor group	*p* value
Gender (M/F)	4/10	4/10	0.0000
Age (years)	58.88 ± 2.401, *n* = 8	65.7 ± 2.765, *n* = 10	0.0891
Present smoker, n (%)	2 (14)	2 (14)	0.0000
Hypertension, n (%)	7 (58)	10 (71)	0.6828
β-Blocker, n (%)	1 (7)	0 (0)	0.0000
Calcium channel blocker, n (%)	0 (0)	5 (36)	0.0407
ACE inhibitors, n (%)	7 (50)	3 (21)	0.2365
Statins, n (%)	4 (29)	5 (36)	0.0000
ALT (U/L)	21.29 ± 2.57	18.83 ± 1.74	0.4527
AST (U/L)	19.43 ± 6.39	19.57 ± 5.39	0.9514
GLU (mmol/L)	5.44 ± 0.11	5.70 ± 0.31	0.0398
GGT (U/L)	28.71 ± 6.46	24.83 ± 5.38	0.0655
TG (mmol/L)	1.50 ± 0.19	1.99 ± 0.18	0.0736
HDL-C (mmol/L)	1.12 ± 0.03	1.15 ± 0.08	0.0005
LDL-C (mmol/L)	2.31 ± 0.14	2.84 ± 0.51	0.3071
CREA (mmol/L)	51.83 ± 2.57	52.28 ± 3.31	0.0001
CK-MB (U/L)	9.71 ± 0.38	9.67 ± 0.67	0.0001
APOA (g/L)	1.24 ± 0.03	1.16 ± 0.03	0.0639
APOB (g/L)	0.75 ± 0.04	0.85 ± 0.10	0.3634
LDH (U/L)	175.70 ± 6.33	178.00 ± 5.60	0.0792

**FIGURE 3:**
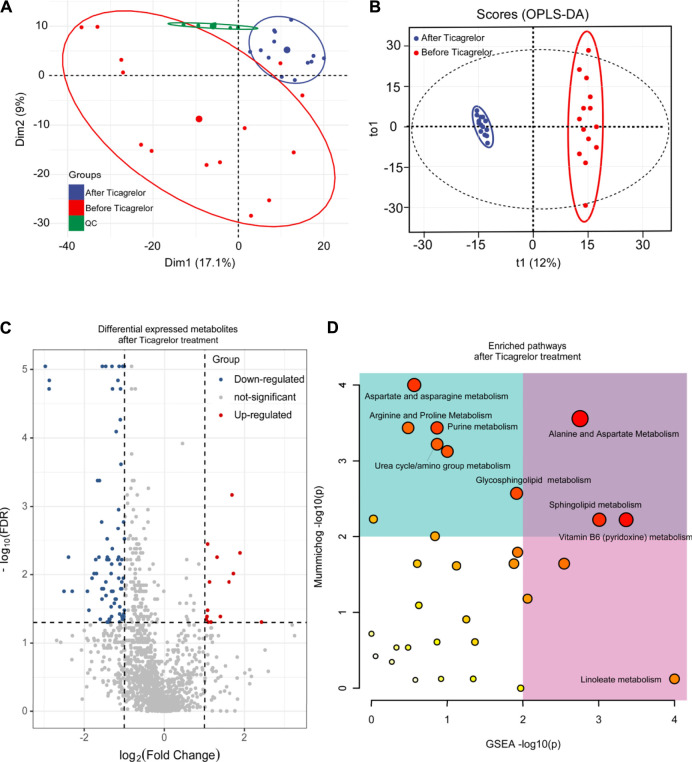
PCA **(A)**, OPLS-DA **(B)**, differential metabolites **(C)**, and differential metabolic pathway **(D)** analysis in the ticagrelor group between before and after treatment.

From the perspective of un-targeted metabolomics, the analysis of human serum samples by LC-HRMS includes two kinds of ions’ (positive and negative) full scan modes. As shown in Figure 3, the total ion chromatograms of the six QC samples were completely overlapped, which indicated that the method had good reproducibility. Based on PCA and OPLS-DA model analysis, LC-MS results were performed to determine the metabolites in the ticagrelor group before and after treatment. From PCA (Figure 3A) and OPLS-DA (Figure 3B) results, the metabolites in the ticagrelor group changed after treatment crucially in contrast to those before treatment. Aggregate fitting parameters, that is, R^2^X and R^2^Y, were 0.271 and 0.981, respectively. And the forecasting parameter, Q^2^, was 0.92. Moreover, about 208 differential metabolites from 1,454 total detected metabolites were screened out according to the loading diagram and VIP value greater than 1 (Figure 3C). The *t*-test for these differential metabolites showed that some of them had significant double-tailed differences (*p* < 0.05). In addition, these compounds were screened by Metline database to obtain the endogenous differential metabolites with exact molecular structure. Finally, these differential metabolites were used to search for corresponding differential metabolic pathways. Alanine and aspartate metabolism, sphingo lipid metabolism, and vitamin B6 metabolism were found as the related metabolic pathway.

**FIGURE 4:**
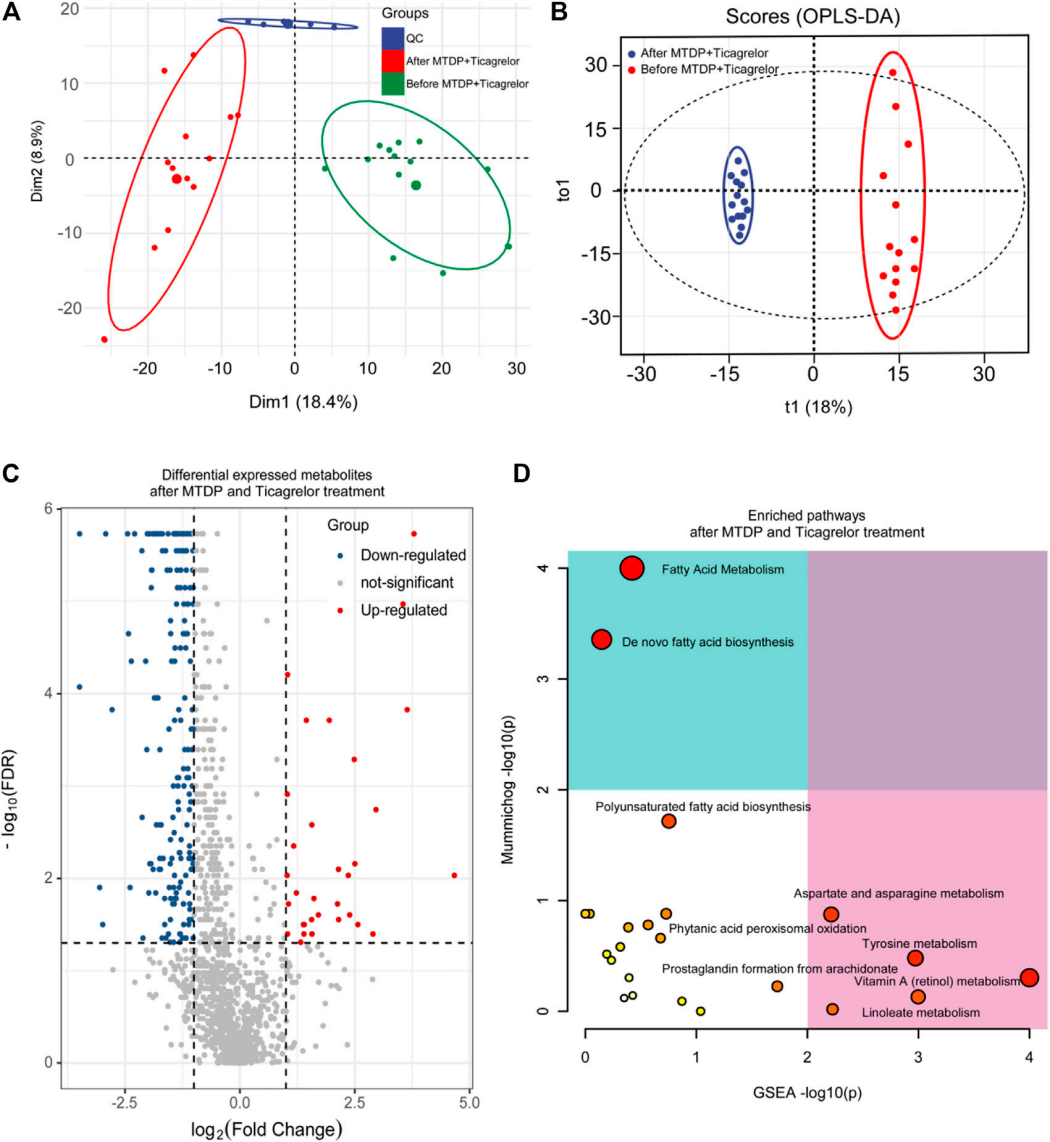
PCA **(A)**, OPLS-DA **(B)**, differential metabolites **(C)**, and differential metabolic pathway **(D)** analysis in the ticagrelor MTDP combination group between before and after treatment.

In addition, based on principal component analysis and OPLS-DA model analysis, differences in metabolites in MTDP and ticagrelor combination between before and after treatment from LC-MS have been identified. From PCA (Figure 4A) and OPLS-DA (Figure 4B) results, the metabolites in the drug combination group changed after treatment crucially in contrast to those before treatment. Aggregate fitting parameters, that is, R^2^X and R^2^Y, were 0.271 and 0.985, respectively. Predictive parameter Q^2^ was 0.876. In addition, according to the load diagram and VIP value greater than 1 (Figure 4C), about 93 differential metabolites were screened out from 1,454 detected metabolites. The *t*-test for these differential metabolites showed that some of them had significant double-tailed differences (*p* < 0.05). In addition, these compounds were screened by Metline database to obtain the endogenous differential metabolites with an exact molecular structure. Finally, these differential metabolites were used to search for corresponding differential metabolic pathways. Aspartate and asparagine metabolism, tyrosine metabolism, linoleate metabolism, and vitamin A metabolism were found as related metabolic pathway.

**FIGURE 5:**
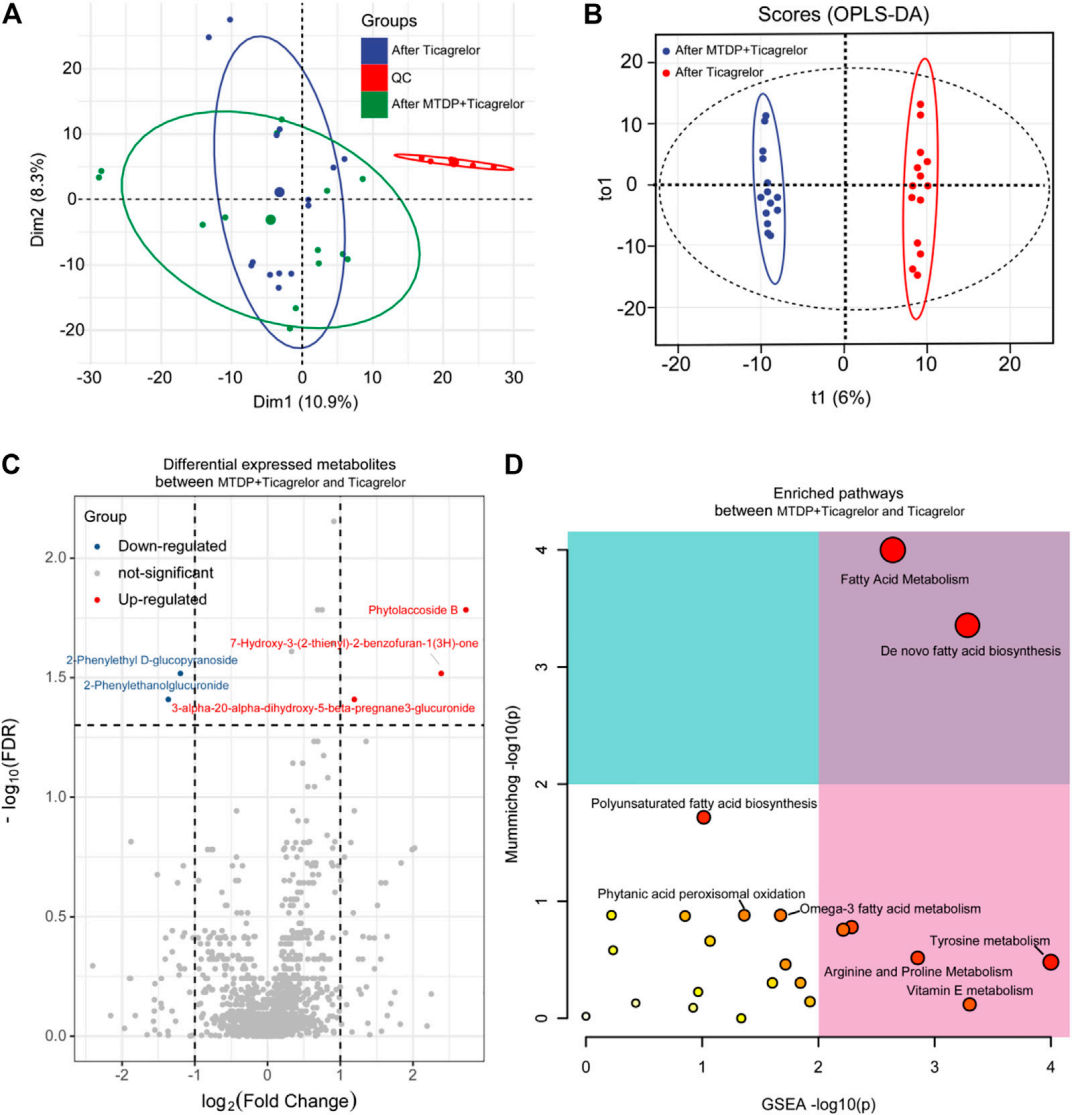
PCA **(A)**, OPLS-DA **(B)**, differential metabolites **(C)**, and differential metabolic pathway **(D)** analysis between the ticagrelor group and the ticagrelor MTDP combination group after treatment.

Moreover, we also did the OPLS-DA analysis between the drug combination group and ticagrelor group after treatment. R^2^Y and Q^2^ were 0.993 and 0.87, respectively. In Figure 5, the two groups were in different quadrants, with significant differences between the combination group and the ticagrelor group. In addition, according to the load diagram and VIP value greater than 1 (Figure 5C), about 93 differential metabolites were screened out from 1,454 detected metabolites. These results indicate that MTDP can significantly enhance the effect of ticagrelor on metabolites *in vivo*. Finally, the metabolic pathways of differential metabolites were analyzed by the MetaboAnalyst web. Fatty acid metabolism was found as the most probable related metabolic pathway.

**FIGURE 6:**
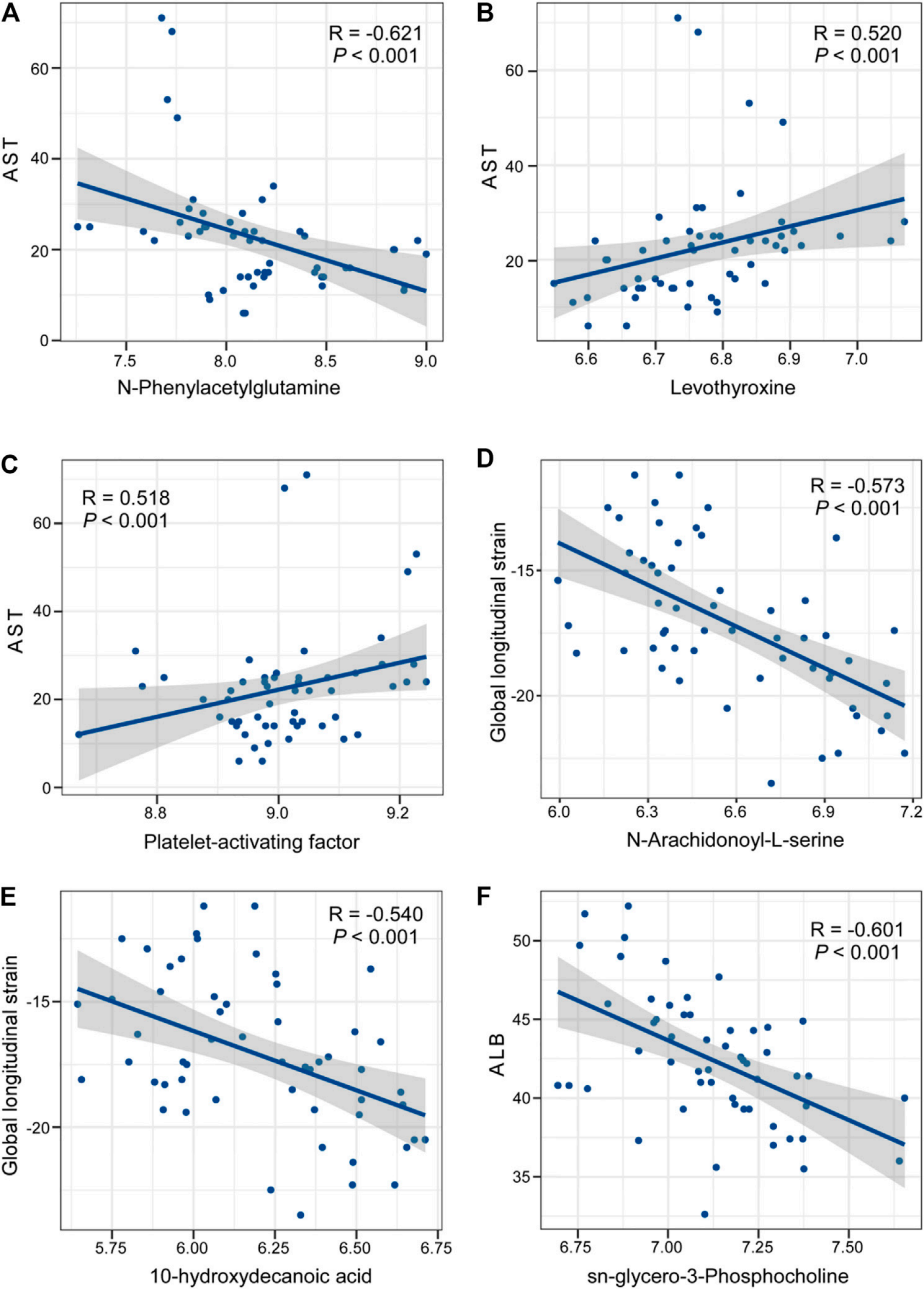
Correlation analysis between differential metabolites in the ticagrelor MTDP combination group and blood biochemical indexes after treatment.

Finally, we compared the blood biochemical indexes in two groups after treatment. From the results (Table 2), several prognostic indicators, such as CK-MB and LDH, in the drug combination group were outstanding lower than those in the ticagrelor group. We also found serum lipid parameters, such as HDL and LDL, in the drug combination group were also much better than those in the ticagrelor group. Then we combined the differential metabolites with these blood biochemical indexes. As shown in Figure 6, N-phenylacetylglutamine, levothyroxine, platelet-activating factor, N-arachidonoyl-l-serine, 10-hydroxydecanoic acid, and sn-glycero-3-phosphocholine had significant correlations with these blood biochemical indexes.

**TABLE 2:** Changes of blood biochemical indexes after treatment in two groups.

Group	Ticagrelor group	Drug combination group	*p* value
ALT (U/L)	28.86 ± 2.27	21.29 ± 2.57	0.0370
AST (U/L)	35.79 ± 16.56	14.33 ± 5.84	0.0004
GLU (mmol/L)	5.44 ± 0.11	5.86 ± 0.24	0.1755
GGT (U/L)	28.71 ± 6.46	34.86 ± 5.50	0.4756
TCH (mmol/L)	3.94 ± 0.22	3.47 ± 0.15	0.0878
TG (mmol/L)	1.50 ± 0.19	1.15 ± 0.08	0.1168
HDL-C (mmol/L)	1.12 ± 0.03	1.27 ± 0.05	0.0222
LDL-C (mmol/L)	2.31 ± 0.14	1.78 ± 0.11	0.0074
CREA (mmol/L)	51.83 ± 2.57	53.29 ± 4.07	0.7643
CK-MB (U/L)	13.29 ± 0.92	9.71 ± 0.38	0.0014
APOA (g/L)	1.24 ± 0.03	1.436 ± 0.04	0.0005
APOB (g/L)	0.75 ± 0.04	0.69 ± 0.02	0.2580
LDH (U/L)	218.60 ± 10.97	175.70 ± 6.33	0.0023

## Discussion

In this study, the mechanism of drug combination therapy was analyzed by using STI combined with the un-targeted metabolism. The pharmacodynamics was evaluated by STI. In addition, the mass spectrometric data were divided satisfactorily between the two groups by PCA and OPLS-DA analysis. In data analysis, we found that 1,454 metabolites differed after treatment between the combination group and the ticagrelor group. These metabolites had dramatical changes, especially in the drug combination group. Finally, we analyzed the metabolic pathways involved according to different metabolites. Fatty acid metabolism had been found as the potential pathway which reflects the drug combination.

Aliphatic acids are the primary components of low-density lipoprotein (Gao et al., 2017). They have relationships with indirectly accumulate monocytes, leading to macrophage proliferation, overexpression of endothelial adhesion factors, and endothelial dysfunction, thereby promoting atherosclerosis (Cui et al., 2017; Paavola et al., 2017). Therefore, lipid metabolism is an independent factor for the evaluation of the prognosis and identification of the mechanism. In our experiment, about six kinds of aliphatic acid, containing FA(20:4), FA(22:5), FA-hydroxy(18:0), palmitic acid, stearic acid, and arachidonic acid, were detected to be lower in the drug combination group, indicating that the MTDP may have the function of lowering blood lipids.

The ingredients of MTDP mainly contain *Salvia miltiorrhiza* Bunge, artificial *moschus*, *Panax ginseng* C.A.Mey, *Venenum bufonis*, artificial *Bovis calculus*, and *Borneolum*, which can reduce the triglyceride and cholesterol in the blood, and inhibit the synthesis of low-density lipoprotein, but also act on a variety of thrombin to inhibit coagulation. Therefore, MTDP can cooperate with ticagrelor in regulating multiple metabolic pathways for the improvement of drug efficacy.

## Conclusion

In this study, we have used the non-targeted metabolomic method based on HPLC-HRMS coupled with STI for the investigation the drug combination mechanism of ticagrelor and MTDP. From the STI analysis, the GLS values increased in the drug combination group, indicating that drug combination can provide better prognosis. The LC-MS results found 93 differential metabolites from 1,454 total detected metabolites in the drug combination group when compared with the ticagrelor group. And the MTDP can affect the lipid metabolism, which have relationship with the blood biochemical indexes after drug treatment. This work has explained the mechanism of the enhancement to ticagrelor by MTDP from the point of view of metabolic product change, and revealed the potential metabolic pathways it affects, which provided the new ideas for clinical medication.

## Data Availability

The original contributions presented in the study are included in the article/Supplementary Material; further inquiries can be directed to the corresponding authors.
